# A Rare Cause of Low Back Pain: A Challenging Diagnosis

**DOI:** 10.7759/cureus.26709

**Published:** 2022-07-10

**Authors:** Tiago Beirão, Jorge Reis, Joana Cochicho, Francisca Costa, Luís Malheiro, Taciana Videira, Joana Pimenta

**Affiliations:** 1 Rheumatology, Centro Hospitalar Vila Nova de Gaia/Espinho, Vila Nova de Gaia, PRT; 2 Internal Medicine, Centro Hospitalar Vila Nova de Gaia/Espinho, Vila Nova de Gaia, PRT; 3 Neuroradiology, Centro Hospitalar Vila Nova de Gaia/Espinho, Vila Nova de Gaia, PRT; 4 Infectious Diseases, Centro Hospitalar Vila Nova de Gaia/Espinho, Vila Nova de Gaia, PRT

**Keywords:** vertebral fractures, muskuloskeletal mri, uropathogenic escherichia coli, septic arthritis of the facet joint, septic arthrits

## Abstract

One of the rarest causes of low back pain is septic arthritis of a lumbar facet joint. We report the case of a 92-year-old diabetic woman with a history of four days of back pain, dysuria, and fever. Due to a sudden worsening of lumbar pain, she went to the emergency department. Physical exam revealed pain with pressure over the D12 vertebral apophyses and the lower-left paraspinal musculature. Laboratory data showed a normochromic normocytic anemia with a hemoglobin of 9.3 g/dL, white cell count of 14.61x10e3/µL (83.1% neutrophils), serum creatinine 1.46 mg/dL and C-reactive protein of 32.11 mg/dL. In urinalysis, nitrites and leukocyturia were identified. CT scan showed an acute D12 fracture and fat stranding at L5, with no irregularities in the discs or in other lumbar spaces. *Escherichia coli* was isolated in blood culture. Lumbar MRI confirmed the diagnosis of septic arthritis of an L5-S1 facet joint and L5 vertebrae osteomyelitis. The patient was successfully treated with intravenous ceftriaxone for eight weeks. As far as we know, this is the second report of septic arthritis of the facet joint caused by *Escherichia coli*.

## Introduction

Low back pain is an important and common health problem, which can be originated from any structure of the spine. One of the rarest conditions is septic arthritis of the facet joint (SAFJ). It is an extremely rare diagnosis, with the first case recorded in 1987 [1]. Since then, only 61 cases have been reported in the literature [2]. This disease has gained increased attention in recent years due to the improvement in imaging techniques. 

This case report describes an elderly diabetic woman who presented to the emergency department with concomitant lumbar fracture, osteomyelitis, and septic arthritis of a lumbar facet joint concomitant with a urinary tract infection. As far as we know, this is the second case report of SAFJ due to *Escherichia coli*.

## Case presentation

A 92-year-old diabetic woman was admitted to the emergency department due to acute intense non-traumatic low back pain. She described a back pain with increasing severity throughout the previous week with acute worsening six hours before admission, that was exacerbated by extension postures. In addition, she reported dysuria, anorexia and fever for four days (highest temperature recorded = 39ºC). She had several comorbidities, namely heart failure, hypertension, type 2 diabetes mellitus (last glycated haemoglobin = 11.6%), chronic kidney disease, and osteoarthrosis. There was no history of corticosteroid or immunosuppressive therapy or previous invasive procedures. 

On admission, her blood pressure was 140/65mmHg, her heart rate was 85 beats per minute, her tympanic temperature was 37.2ºC, and oxygen saturation level (pulse oximetry) was 97% without supplementary oxygen. No neurologic impairment, dehydration signs, skin abnormalities, or lymphadenopathies were observed. Cardiac and pulmonary physical examinations were normal, except for a grade II mild systolic murmur in the mitral area. The abdominal physical examination was normal. There was pain with palpation of the D12 vertebral apophysis and on the lower-left paraspinal musculature. No abnormalities were detected in sensitivity, strength, and osteotendinous reflexes of the lower limbs.

**Figure 1:**
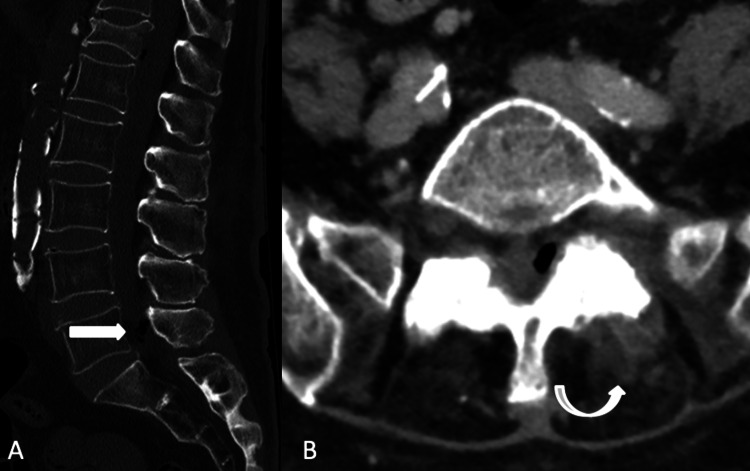
The initial assessment scan depicted the D12 vertebral body compressive fracture and revealed the presence of air foci in the epidural space (A) adjacent to the L5-S1 intervertebral joint, associated with thickening of local soft tissues (B).

**Figure 2:**
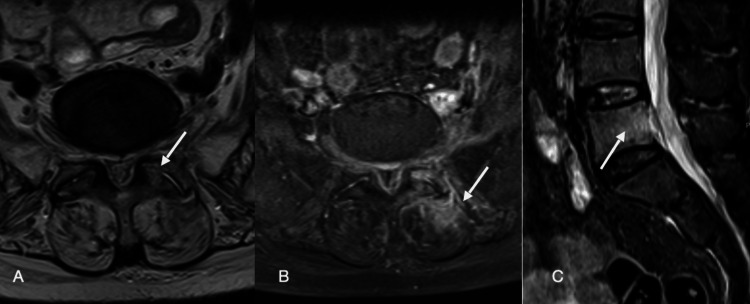
Images depicting the septic arthritic process in (A) axial T2 WI with poor differentiation of the synovial space, corresponding to (B) intense local enhancement with epidural and muscular involvement, shown on the axial fat-suppressed DIXON images. Also worth noticing, (C) sagittal STIR images with signs of osteomyelitis of the vertebral L5 body.

Laboratory data revealed a normochromic normocytic anemia (hemoglobin - 9.3 g/dL), leucocytosis (leucocytes - 14.61x10e3/µL) with neutrophilia (83.1%) and an increased serum C-reactive protein level (32.11 mg/dL; normal range 0-5 mg/dL); platelet count (258 x10e3/µL) and coagulation tests were normal. The serum glucose level was 199 mg/dL. Serum creatinine was 1.46 mg/dL (previous 1.06 mg/dL) with urea of 83 mg/dL. Serum electrolyte and liver parameters were normal. Urinalysis revealed nitrites and leukocyturia. Electrocardiogram and chest X-ray were normal. Lumbar Spine Computer Tomography (CT) scan revealed acute D12 fracture (Figure 1) and fat stranding at L5, with no irregularities of the discs or other lumbar spaces. There was no evidence of valve vegetations on the transthoracic echocardiogram. Urine and blood cultures were collected. Due to clinical suspicion of lumbar bone involvement, empirical antimicrobial therapy with intravenous ceftriaxone 2g every 12 hours was started immediately. *Escherichia coli* was isolated in 4:4 blood cultures. Lumbar Magnetic Resonance Imaging (MRI) showed L5 osteomyelitis, S1-L5 arthritis of the left lumbar facet joint, and epidural empyema (Figure 2).

Clinical and laboratorial improvement was observed after 11 days with ceftriaxone therapy. Blood cultures were negative after 48 hours of antimicrobial therapy. A conservative orthopaedic approach to the D12 fracture was selected, with lumbar orthosis and analgesia. Ceftriaxone was maintained for eight weeks according to the antibiogram. 

**Figure 3:**
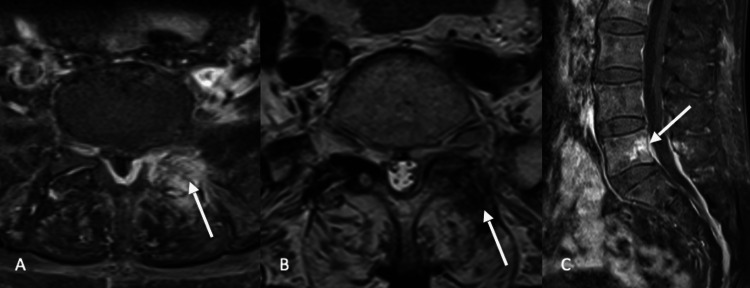
The 3-month follow-up scan revealed (A) continued local post-contrast enhancement with decreased epidural empyema with (B) decreased synovial differentiation while the vertebral body osteomyelitis remained local (C).

After eight weeks of therapy, the lumbar MRI was repeated and a decrease in the epidural empyema and paraspinal muscles edema was observed (Figure 3). The patient had full clinical resolution of musculoskeletal manifestations and normalisation of the inflammatory parameters.

## Discussion

Most SAFJ cases occur during the sixth decade of life, with no gender dominance [3]. The oldest patient with SAFJ recorded until this case was diagnosed at the age of 83 years [4]. The lumbar region is the most commonly affected area in SAFJ, with L4-L5 being the most frequently involved joint [5]. In a recent review of 11 SAFJ cases,only two patients had infection on the L5-S1 joint [5]. Just like in our case, most case reports describe unilateral involvements of the facet joint [4].

Several risk factors have been associated with SAFJ. Factors that lead to an immunocompromised state are usually present in clinical history, with at least one present in 38% of cases [4]. Underlying neoplastic disease, end stage-renal failure, alcoholism, intravenous drug abuse, and diabetes have been described in patients with SAFJ [6,7]. Our patient had two factors: history of poorly controlled type 2 diabetes, with a HbA1c of 11.2% and chronic kidney disease.

An etiologic diagnosis is usually based on blood cultures and percutaneous drainage. Surgical samples collection is reserved for when less invasive tests fail to identify the cause or when surgical debridement is needed. SAFJ is usually associated with a monomicrobial infection, with only a few polymicrobial infections reported [4]. *Staphylococcus aureus* is responsible for the majority of cases (70%), followed by *Streptococcus* species in 16% of the cases [8]. *Escherichia coli* is an utmost infrequent agent, with only one case reported in the literature [9].

The other case of SAFJ associated with *Escherichia coli* infection previously described occurred in a 77-year-old diabetic woman who developed back pain after being diagnosed with a genitourinary infection [9]. The clinical and laboratory data were similar to this case and the patient was treated with intravenous cefotaxime and gentamicin for 21 days with complete recovery.

Although the precise pathophysiology of the infection spread is unclear, it seems that it can occurs by three pathways [10]. The most probable is an hematogenous spread with bacteremia originating from a distant site infection. The lower blood supply of the posterior area in comparison with the blood supply of the vertebral marrow could explain the reduced probability of infection and the low prevalence of SAFJ compared to spondylodiscitis [9]. The second mechanism is a direct inoculation of the facet joint after a mechanical strain of the facet joint that can cause an effusion and lead to bacterial colonisation [1]; and, finally, a third mechanism is by contiguous spread from an adjacent infection of the muscle, disc, and/or vertebral body [10]. 

In our case, several mechanisms could be involved. The presence of fever, dysuria, and nitrites and leukocyturia in urinalysis suggests an hematogenous spread from the urinary tract. Additionally, the existence of osteomyelitis present in L5 could contribute to the contiguous spread of the infection. 

From a clinical point of view, patients usually present with an unilateral acute or subacute lateralized inflammatory back pain, not relieved by rest, which can be radicular and associated with neurological deficits [11]. These symptoms are similar to those observed in spondylodiscitis, so imagining techniques are the key to differential diagnosis between these pathologies. Our clinical case presented with urinary symptoms and back pain, common manifestations at the emergency department. However, increased pain at palpation of vertebral apophysis and unilateral paraspinal musculature raised clinical suspicion of bone/joint involvement, which led to ordering a lumbar spine CT scan. The presence of fat straining in L5 increased the clinical suspicion and, later, the MRI confirmed this rare diagnosis. In the present case, the coexistence of a spontaneous fracture in D12, probably of osteoporotic nature, explains the unusually acute back pain presentation. 

Different analytic abnormalities have been reported in SAFJ cases; 50% showed leucocytosis and all, except two cases, presented with increased erythrocyte sedimentation rate and/or C-reactive protein level [2,12]. Both abnormalities were present in our case report. Blood cultures are positive in the majority of cases [10]. 

Imaging is the basis for the diagnosis of SAFJ. Plain radiography has low sensitivity and remains normal at the acute onset of symptoms [13]. CT scans have higher sensitivity but low specificity for SAFJ [13]. MRI is the most sensitive and specific technique and it is considered the gold standard for SAFJ diagnosis and follow-up [13]. Technetium-99 scan can also be used and has 100% sensitivity, but it is not required for most cases [5]. 

The treatment of SAFJ is antimicrobial therapy with additional surgical intervention being rarely needed and usually reserved when severe neurological compromise is present or the infection is refractory to antimicrobials [1,14]. Antibiotics with good penetration profiles in bone and joints represent potential options for the treatment of osteomyelitis and septic arthritis. Most cases require six to eight weeks of treatment [14]. In our case, the patient was treated with ceftriaxone for eight weeks due to the presence of epidural empyema. Bed rest and orthosis can be used for pain management, and this was particularly important for our patient, due to the concomitant presence of a lumbar fracture [4].

## Conclusions

In conclusion, SAFJ is an exceptionally rare diagnosis, with increased reports in recent years due to better diagnostic techniques. Despite this, SAFJ is not always a straightforward diagnosis. In our case, the concomitant presence of urinary tract infection, osteomyelitis, and acute lumbar fracture contributed to the atypical presentation. Nonetheless, clinical history and physical examination should be the basis for diagnosis suspicion, with imaging techniques needed for confirmation. A multidisciplinary approach is also important since SAFJ can be associated with several organ system disorders, may require surgical intervention and benefits from antimicrobial stewardship.
